# The health-related quality of life of children with multiple sclerosis is mediated by the health-related quality of life of their parents

**DOI:** 10.1177/13524585211061521

**Published:** 2022-02-07

**Authors:** Julia O’Mahony, Brenda Banwell, Audrey Laporte, Adalsteinn Brown, Lady Bolongaita, Amit Bar-Or, E Ann Yeh, Ruth Ann Marrie

**Affiliations:** Department of Internal Medicine, University of Manitoba, Winnipeg, MB, Canada; Division of Child Neurology, The Children’s Hospital of Philadelphia, Perelman School of Medicine, University of Pennsylvania, Philadelphia, PA, USA; Canadian Centre for Health Economics, Toronto, ON, Canada/Institute of Health Policy, Management and Evaluation, University of Toronto, Toronto, ON, Canada; Dalla Lana School of Public Health, University of Toronto, Toronto, ON, Canada; Canadian Centre for Health Economics, Toronto, ON, Canada/Institute of Health Policy, Management and Evaluation, University of Toronto, Toronto, ON, Canada; Center for Neuroinflammation and Experimental Therapeutics and Department of Neurology, Perelman School of Medicine, University of Pennsylvania, Philadelphia, PA, USA; Department of Pediatrics, University of Toronto, Toronto, ON, Canada/Division of Neurology, Neurosciences and Mental Health, The Hospital for Sick Children, SickKids Research Institute, Toronto, ON, Canada; Departments of Medicine and Community Health Sciences, Max Rady College of Medicine, Rady Faculty of Health Sciences, University of Manitoba, Winnipeg, MB, Canada

**Keywords:** Demyelination, CIS, multiple sclerosis, outcome measurement, quality of life

## Abstract

**Background:**

We previously found that children with the chronic disease multiple sclerosis (MS) reported lower health-related quality of life (HRQoL) when compared to children who experienced the transient illness termed monophasic acquired demyelinating syndromes (monoADS). Parents of children with MS also reported lower HRQoL.

**Objectives:**

We evaluated whether parental HRQoL mediated the relationship between the diagnosis of MS and the HRQoL of affected children. To ascertain the effect of an MS diagnosis, we compared children with MS to those with monoADS.

**Methods:**

Children were enrolled in a prospective multi-site Canadian study. Random effects models evaluated whether parental HRQoL mediated the relationship between the diagnosis of MS and the HRQoL of affected children, adjusting for child and family characteristics.

**Results:**

207 parent-child dyads (65 MS; 142 monoADS) completed HRQoL questionnaires. When we modeled the child’s HRQoL adjusting for covariates, but not the parent’s HRQoL, the diagnosis of MS associated with lower HRQoL of the child (*p* = 0.004). When we added parental HRQOL to the model, the association between the diagnosis of MS and the child’s HRQoL diminished (*p* = 0.13).

**Conclusions:**

Parental HRQoL mediated the relationship between the diagnosis of MS and the HRQoL of affected children, emphasizing the importance of family-centered care.

## Introduction

We, and others, have found that children diagnosed with the chronic neurological disease multiple sclerosis (MS) reported lower health-related quality of life (HRQoL) when compared to children who experienced the transient neurological illness termed monophasic acquired demyelinating syndrome (monoADS), healthy controls, and siblings.^1–4^ The cause of lower HRQoL, specifically emotional functioning, among children with MS in our prior study is not obvious because most children in our cohort had no neurological impairments that limited functional activities and appeared similar to their healthy peers.^1^ However, parents of children with MS reported lower HRQoL in every domain of the PedsQL^TM^ Family Impact Module when compared to parents of children who experienced monoADS, even when their children had neither physical impairments nor relapses.^1^ These findings implicate an effect of the diagnosis of MS on the HRQoL of parents of affected children. This is consistent with parents describing their experiences with their child’s MS diagnosis as being dominated by feelings of uncertainty.^5^ Long-term outcomes of pediatric-onset MS are unpredictable, including the effects on educational outcomes, labor force participation, and likelihood of progressive disease. Our prior work evaluated the HRQoL among children with MS and separately evaluated the HRQoL of their parents, but did not evaluate the HRQoL interplay within parent–child dyads facing pediatric-onset MS.

The HRQoL of parent and child are associated in other pediatric-onset chronic conditions with unpredictable heterogeneous outcomes. Among families facing epilepsy and cancer remission during childhood, parental psychosocial dysfunction was associated with lower HRQoL of the child.^6,7^ Parental depression mediates the effect of inflammatory bowel disease (IBD) activity on the child’s HRQoL.^8^ Parents with depressive symptoms are hypothesized to have difficulty facilitating appropriate communication and coping techniques for their children with IBD, leading to lower emotional functioning among children.^8^

**Figure 1.**
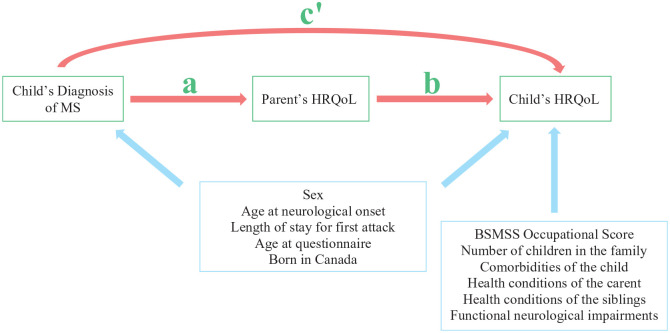
Conception of hypothesized mediation of the child’s HRQoL by the parents’ HRQoL. We hypothesized that the HRQoL of parents would mediate the relationship between the child’s diagnosis of MS and the child’s HRQoL. We expected to observe an association between that diagnosis of MS and the child’s HRQoL (effect of diagnosis on child’s HRQoL represented by c’). The effect between the diagnosis and the mediator (parent’s HRQoL) is represented by a. The effect between the mediator and the child’s HRQoL is represented by b. We hypothesized that some covariates would associates with both the child’s diagnosis and the child’s HRQoL while other covariates would associate with only the child’s diagnosis.

We evaluated whether parental HRQoL mediated the relationship between the diagnosis of MS and the HRQoL of children with MS in a prospective cohort study. To control for the diagnosis of MS, we compared children with MS to a group of children with monoADS who were followed concurrently. Children with MS and monoADS shared the experience of an acute neurological illness, but children with MS and their families learned of the risk for more attacks and the long-term implications of an MS diagnosis. As such, monoADS and MS patients and families diverge in terms of their lived experiences and expectations. We hypothesized that the HRQoL of parents would mediate the effect of the child’s MS diagnosis on the child’s HRQoL (Figure 1). Such findings might implicate parental HRQoL as a means of improving the HRQoL of children with MS.

## Methods

### Participant enrollment and follow-up

Between September 2004 and May 2013, we enrolled participants aged <16 years with MS or monoADS within 90 days of their first clinical neurological signs (herein termed neurological onset) at one of 23 Canadian health care facilities.^9,10^ Between August 2015 and January 2018, we enrolled additional participants aged <18 years, within 180 days of neurological onset at two sites. Relapses were defined as new neurological impairments persisting >24 hours in the absence of acute fever or illness, and confirmed by examination. MonoADS was defined by the absence of relapses or new lesions on serial brain MRI.^9,10^ Study visits occurred at study enrollment, 3, 6, and 12 months after neurological onset, and annually thereafter.^1^

Given our objective to evaluate the HRQoL of children with MS and their parents, we excluded participants with neuromyelitis optica or relapsing demyelination who did not meet the diagnostic criteria for MS (including those with relapsing illness with antibodies to myelin oligodendrocyte glycoprotein).

Ethics approval was obtained at all sites. Parents or legal guardian(s) (herein termed parents) signed informed consents and younger children provided assent.

### Health-related quality of life (HRQoL)

Beginning in 2010, HRQoL was ascertained at all study visits >30 days from neurological onset or relapses (MS participants) using the PedsQL^TM^ Inventory (Child Report) and Family Impact Module (see Supplementary Methods). These tools evaluated the participant’s self-reported HRQoL ( $HRQoL_{it}^{child}$ ), the parent’s (mother or father, one parent per participant, not necessarily the same parent evaluated at each assessment) self-reported HRQoL ( $HRQoL_{it}^{parent}$ ), and the parent’s report of their family’s functioning ( $HRQoL_{it}^{family\leftarrow parent}$ ). Questionnaires were evaluated according to the PedsQL^TM^ scoring guidelines with higher scores indicating better HRQoL (100 best health; 0 worst health).^11^ Dimensional, subscale, and overall scores can be computed for the PedsQL^TM^; analyses were restricted to the overall scores to limit multiple comparisons. Participants were required to have contemporaneously (within 30 days) completed the PedsQL^TM^ Inventory (Child Report) and PedsQL^TM^ Family Impact Module, including the Parent HRQL Summary Score and the Family Functioning Summary Score, at ⩾1 study visit(s) to be included in this analysis. Modeling of the repeated HRQoL assessments is described below.

### Neurological examination

Neurological findings were evaluated and recorded at study visits by trained investigators using a descriptive scale of clinical severity capturing the eight functional systems assessed by the Expanded Disability Status Scale (EDSS).^12^ For this analysis, participants were considered to have normal or mild neurological impairment (EDSS ⩽ 2.5) if they had normal gait, no encephalopathy, had corrected visual acuity better than 20/30 bilaterally, did not experience difficulties with bowel or bladder control, had normal or minimal pyramidal dysfunction, and normal or mild decreases in sensory function in <3 limbs. Neurological function was assessed concurrently with HRQoL.

### Barratt Simplified Measure of Social Status (BSMSS) Occupation Score

Social status was ascertained using the Barratt Simplified Measure of Social Status (BSMSS) Occupation Score because socio-economic status is related to the well-being, mental health, and physical health of children.^13,14^ The BSMSS is based on employed status and occupational prestige of each child’s parents,^15^ and assigns a score ranging from 5 to 45 with higher scores indicating higher social status.^16^ Ascertainment of parental occupation is detailed in Supplementary Methods. The child’s country of birth was included as a covariate to account for the sizable proportion (43%) of internationally educated immigrants in Canada who report working in occupations for which they are over-qualified.^17^ Parents completed the BSMSS at their first study visit after it was introduced to the study protocol in 2015. We modeled BSMSS as a time-invariant variable in the multivariable models.

### Comorbidities and health conditions

A modified version of a comorbidity count scale was used to capture the number of comorbidities experienced by participants and the number of health conditions experienced by their siblings (all siblings combined) and parents (combined), detailed in Supplementary Methods.^18,19^ We controlled for the number of health conditions among parents when estimating the child’s HRQoL because parental illness is associated with the psychosocial health of children.^20^All siblings (full, half, and step) were considered equal irrespective of cohabitation status. The number of comorbidities and health conditions seldom changed over the period of observation so they were included as time-invariant variables in the multivariable models.

### Analyses

Participant characteristics are described using median (interquartile range (IQR)) for continuous variables and frequency (percent) for categorical variables. We compared the MS versus monoADS groups with respect to the dependent and independent variables selected for inclusion in each of the models described herein; χ^2^, Wilcoxon, or Kruskal–Wallis tests were performed as appropriate accounting for clustering of repeated measures at the individual level by using the average value for each participant. Bivariate analyses were also performed with respect to the HRQoL of the child ( $HRQoL_{it}^{child}$ ), accounting for clustering at the individual level using random effects specifications to account for within-individual effects that may vary over time.^21^

We constructed four multivariable regression models using random effects specifications to evaluate whether parental HRQoL mediated the relationship between the diagnosis of MS (versus monoADS) and the HRQoL of affected children.^22^ Random effects methods account for the correlated nature of repeated HRQoL measures within participants by modeling and partitioning the covariance structure of the outcomes within and between participants, allowing for calculation of the variance that is due to within-participant variation versus that due to between-participant variation.^23^ These models accommodate variable numbers of HRQoL observations per participant and allow for adjustment of time-invariant factors that do not change within participants over the period of HRQoL observations and time-variant factors that fluctuate between HRQoL assessments.

Changes in the parent’s HRQoL may be accompanied by changes in the child’s HRQoL and vice versa, termed reverse causality, which could lead to biased estimates in the multivariable regression model.^24^ Tests for reverse causality of the key predictor ( $HRQoL_{it}^{parent}$ ) in our model were affirmative.^24^ The HRQoL of the family from the parent’s perception ( $HRQoL_{it}^{family\leftarrow parent}$ ) was therefore enlisted as an instrumental variable to generate predictive values of the HRQoL of the parent ( $\hat{HRQoL_{it}^{parent}}$ ). Instrument validity and strength was evaluated using the Stock-Yogo test.^25^ Independent variables were evaluated for multicollinearity.

Mediation was evaluated using the following four equations:^22^

Regression of the dependent variable on the independent variable

(1) $$HRQoL_{it}^{child}=MS+X_{it}+X_{i}+\mu_{it}+\varepsilon_{it}$$

Regression of the mediator on the independent variable

(2) $$\hat{HRQoL_{it}^{parent}}=MS+X_{it}+X_{i}+\beta_{it}+{Þ}_{it}$$

Regression of the dependent variable on the mediator

(3) $$HRQoL_{it}^{child}=\hat{HRQoL_{it}^{parent}}+X_{it}+X_{i}+\gamma_{it}+\zeta_{it}$$

Regression of the dependent variable on the mediator and the independent variable

(4) $$HRQoL_{it}^{child}=\hat{HRQoL_{it}^{parent}}+MS+X_{it}+X_{i}+t_{it}+{Ζ}_{it}$$

where $HRQoL_{it}^{child}$ is the HRQoL of the child from the child’s perception; $\hat{HRQoL_{it}^{parent}}$ is the predictive value of the HRQoL of the parent from the parent’s perception; MS is the diagnosis of MS (monoADS as reference group; binary); $X_{it}$ is the time-variant independent variables; $X_{i}$ is the time-invariant independent variables; and $\mu_{it},\beta_{it},\gamma_{it},\zeta_{it}$ are between-individual error; and $\varepsilon_{it},{Þ}_{it},t_{it},z_{it}$ are within-individual error.

The time-invariant covariates ( $X_{i}$ ) included sex (male as reference group, binary); age at onset (years, continuous); length of stay in hospital for first attack (days, continuous); born in Canada (born outside Canada as reference group, binary); BSMSS Occupation Score (ordinal); number of siblings (count); comorbidities of the participant (count); health conditions of the participant’s parents (count); and health conditions of the participant’s siblings (count). Time-variant covariates ( $X_{it}$ ) included the participant’s age at the time of HRQoL assessment (years; continuous) and presence of functional neurological impairments (normal or mild impairments as reference group; binary).

The multivariable modeling methods exclude participants for whom covariates are missing so variables unique to children with MS (e.g., relapses and exposure to disease-modifying therapies (DMTs)) were not included. Relapses and DMT exposure were recorded at each visit and are reported to inform on the generalizability of our findings.

To evaluate whether the relationship between parent and child HRQoL changed across HRQoL observations, we constructed a fifth regression model by adding an interaction term between parental HRQoL and study visit number in equation (4).

Statistical analyses were performed using Stata version 15.1.^26^

## Results

### Participants

**Figure 2.**
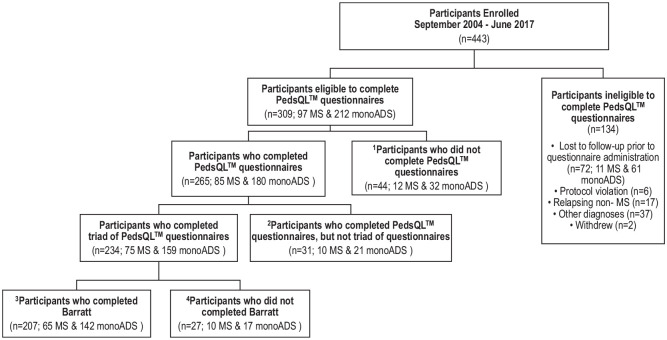
Participants enrolled with pediatric-onset multiple sclerosis (MS) and monophasic acquired demyelinating syndromes (monoADS). Between September 2004 and January 2018, 443 children were enrolled, of whom 309 were eligible for the present analysis. Of these, 44 declined to complete the HRQoL questionnaires leaving 265 families who completed at least one of the three questionnaires (Child Inventory, Parent HRQoL, and Family HRQoL). Of the 234 families who completed contemporaneously matched triads of questionnaires, 207 also completed the BSMSS Occupation Scale and were included in these analysis. Among the 207 unique families included in this analysis, 1,863 questionnaires from 621 time points were included.^1^ Ten participants submitted incomplete questionnaires (2 Child Inventories and 8 Family Impact Modules) and were therefore excluded from the current analyses. These participants are categorized in Figure 2 as not having completed the questionnaires.2 31 unique families were not included in this analysis because they did not complete the Child Inventory, Parent HRQoL, and Family HRQoL (triad) at a single time point. These 31 unique families did however complete 51 questionnaires at 42 unique time points.3 27 unique families were not included in these analyses because they did not complete the BSMSS Occupation Scale. These 27 unique families did however complete 175 questionnaires at 67 unique time points (triads were completed at 48 of the 67 time points). Twelve of these participants also submitted incomplete Family Impact Modules at a single time point; the incomplete Family Impact Modules and the completed Child Inventories were disregarded at those time points.4 Among the 207 unique families included in these analyses, 151 questionnaires from 110 unique time points were excluded from this analysis because they were not contemporaneously matched within a triad (Child Inventory, Parent HRQoL and Family HRQoL). Two participants submitted incomplete Child Inventory Modules and were therefore excluded and categorized as having not completed the questionnaires. Participants included in this analysis (*n* = 207) did not differ (*p* > 0.05) from those who were eligible to complete the questionnaire and were excluded (*n* = 102) from this analysis (either because the participant declined to complete or did not complete a contemporaneously matched triad and BSMSS) in terms of MS diagnosis, age at onset, and sex. Participants who were included in the current analyses had a longer length of follow-up (median (IQR) 7.24 (5.18–9.05) years) than those who were not included (5.46 (3.09–7.45) years; *p* < 0.0001).

Between September 2004 and January 2018, 443 children were enrolled, of whom 207 (65 MS; 142 monoADS) were eligible for the current analysis (Figure 2). The 207 eligible participants and their parents completed the BSMSS and contemporaneously matched triads of the child-parent-family HRQoL modules at 621 time points 5.11 (3.09–7.24) years after neurological onset. Participants completed a median (IQR; min-max) of 4 (3–6; 1–8) questionnaires over 3.94 (1.71–5.11) years. Functional neurological impairments (EDSS > 2.5) were documented in 30 of 207 (14%) participants (14 MS, 16 monoADS; Table 1).

**Table 1.** Characteristics of the cohort.

Characteristic	monoADS (*n* = 142)	MS (*n* = 65)	*p values*
Characteristics at neurological onset			
Female, *n* (%)	64 (45)	43 (66)	**0.005**
Age at neurological onset (years), median (IQR), min—max	8.67 (4.62–12.34) 0.46–15.89	14.49 (11.75–15.72) 1.90–17.86	**<0.001**
Length of stay for first attack (days), median (IQR)	7 (5–11)	3 (0–7)	**<0.001**
Participant born in Canada, *n* (%)	130 (92)	52 (80)	**0.02**
^a^Parental BSMSS Occupation Score	27.5 (17.5–35)	25 (12.5–35)	0.34
Number of children in the family, median (IQR)	2 (2–3)	2 (2–3)	0.74
Characteristics at first HRQoL assessment			
Age at questionnaire (years), median (IQR), min—max	12.99 (8.86–16.34) 5.35–25.22	17.19 (14.85–19.52) 7.92–28.39	**<0.001**
^b^Persistent neurological impairments present without recovery, *n* (%)	13 (9)	3 (5)	0.05
^b^Transient neurological impairments present that subsequently resolved, *n* (%)	3 (2)	11 (17)	0.06
Disease duration (years), median (IQR)	4.15 (3.02–6.09)	3.13 (0.46–6.05)	**0.02**
^c^Comorbidities of the participant, n (%)	25 (18)	14 (22)	0.56
^d^Health conditions of the parent, *n* (%)	57 (40)	33 (51)	0.62
^e^Health conditions of the participant’s siblings, *n* (%)	15 (11)	8 (12)	0.93
^f^Characteristics over period of HRQoL assessments			
Child’s age at questionnaire (years), median (IQR)	14.40 (11.08–6.15) 5.35–24.93	17.87 (16.25–20.24) 7.01–26.47	**<0.001**
Functional neurological impairments, median (IQR)	0 (0–0)	0 (0–0)	0.06
Disease duration (years), median (IQR)	5.69 (4.01–7.38)	4.73 (1.02–7.16)	**0.02**
PedsQL^TM^ HRQoL results			
Number of questionnaires per participant, median (IQR)	3 (2–4)	2 (2–4)	0.77
^g,h^Health-related quality of life of the child ( $HRQoL_{it}^{child}$ )	86.25 (77.17–92.66)	79.12 (65.22–89.86)	**0.002**
^g^Health-related quality of life of the parent ( $HRQoL_{it}^{parent}$ )	89.58 (78.58–97.29)	78.13 (62.92–92.12)	**<0.001**
^g^Health-related quality of life of the family from the parent’s perspective ( $HRQoL_{it}^{family\leftarrow parent}$ )	92.19 (79.69–97.27)	73.83 (55.47–85.94)	**<0.001**

monoADS: monophasic acquired demyelinating syndromes; MS: multiple sclerosis; IQR: interquartile range; BSMSS: Barratt Simplified Measure of Social Status; HRQoL: health-related quality of life. *P* values < 0.05 were considered statistically significant and presented in bold text.

a Parental BSMSS Occupation Score is ordinal and varies from 5 (lowest level of occupation) to 45 (highest level of occupation).

b Among the 65 MS participants, three (5%) experienced persistent neurological impairments without recovery; one child experienced ambulatory impairments, but did not require mobility aid, and two experienced permanent uniocular visual loss. Eleven (17%) children with MS were experienced residual neurological impairments following relapses that occurred greater than 30 days prior to HRQoL observation that subsequently resolved (two sensory, two ambulatory who did not require mobility aid, four ambulatory who required mobility aid, one visual, and one pyramidal) and one child experienced two discrete transient episodes of visual and pyramidal dysfunction. Among the 142 monoADS participants, 13 experienced persistent neurological impairments without recovery following their sole episode of demyelination, including three children with bowel or bladder dysfunction, one child with ambulatory impairments who is wheelchair dependent, two children with ambulatory impairments who required mobility aid, one child with ambulatory impairments who did not require mobility aid, one child with pyramidal dysfunction, one child with sensory dysfunction, one child with moderate upper limb hemiparesis, and three children with visual impairments.

c 25 unique participants with monoADS and 14 unique participants with MS reported one or more comorbidities.

d 57 unique sets of parents of children with monoADS and 33 unique sets of parents of children with MS reported one or more health conditions.

e 15 unique families of children with monoADS and 8 unique families of children with MS reported one or more sibling with at least one health condition.

f Average per participant over period of observation (between first HRQoL assessment to most recent HRQoL assessment).

g PedsQL^TM^ Questionnaire scores are continuous from 0 (worst health) to 100 (best health).

h The mean (SD) HRQoL of children with MS aged 8 to 18 was 79.68 (15.06) and was 82.25 (13.34) for children with monoADS. Notably, these values fall within one SD of the mean of a large school-aged population of healthy children (*n* = 3,990; mean (SD) 81.08 (13.07)) and those experiencing chronic illness (*n* = 201; mean (SD) 71.59 (16.17)) within the same age range.^27^

The median BSMSS Occupation Score was 25 for MS participant families and 27.5 for the families of the monoADS participants (Table 1). Of the 207 participating families, 27 (13%) reported an occupation status for one parent and 180 (87%) reported occupation statuses for both parents. Cohabitation status of parents was not queried.

Over half of the participating families reported ⩾1 family member with a health condition, in addition to the demyelinating disease of the child (Table 2). Twenty-seven families reported one or more members of the child’s immediate family with ⩾1 psychiatric diagnosis (anxiety, depression, and bipolar disorder), including 15 children with MS or monoADS who reported 19 co-morbid psychiatric diagnoses (Table 2).

**Table 2.** Comorbidities and health conditions of participant families.

Condition	Number of affected family members					
	Participants			^a^Sibling	^b^Mother	^b^Father
	^c^MS participant	^d^monoADS participant	Combined MS and monoADS			
Bipolar disorder	3	0	3	4	2	2
Anxiety disorder	3	7	10	1	2	1
Depression	0	6	6	3	7	2
Hyperlipidemia	0	0	0	0	2	4
Hypertension	0	0	0	1	4	7
Heart trouble	0	0	0	0	0	3
Diabetes mellitus	0	0	0	1	6	13
Cancers	0	2	2	1	7	5
Glaucoma	0	0	0	0	1	0
Thyroid disease	2	3	5	2	19	3
Lupus	0	0	0	0	4	1
Inflammatory bowel disease (IBD)	0	1	1	1	6	1
Irritable bowel syndrome (IBS)	0	3	3	0	0	1
Epilepsy	2	3	5	2	2	1
Migraine	2	4	6	2	2	1
Rheumatoid arthritis	1	0	1	0	5	0
Blood disease	0	0	0	1	1	1
Kidney disease	0	1	1	0	2	1
Lung trouble	0	0	0	0	1	0
Open ulcer of the stomach	0	0	0	0	1	0
Osteoporosis	0	0	0	0	1	0
Guillain–Barré Syndrome (GBS)	1	0	1	0	0	1
Tics	1	2	3	0	0	0
Endocrine disorder	1	1	2	0	0	0
Parkinson’s disease	0	0	0	0	0	1
Central nervous system (CNS) aneurysm	0	0	0	0	0	1
Autism spectrum disorder (ASD)	0	0	0	4	0	0
Multiple sclerosis (MS)	0	0	0	2	2	1
Myasthenia gravis (MG)	0	0	0	0	1	0
Sarcoidosis	0	0	0	0	0	1
Usher’s syndrome	0	1	1	1	0	0
Total	16	34	50	26	78	52

monoADS: monophasic acquired demyelinating syndromes; MS: multiple sclerosis; IBD: inflammatory bowel disease; IBS: Irritable bowel syndrome; GBS: Guillain-Barré syndrome; CNS: Central nervous system; ASD: Autism spectrum disorder; MG: Myasthenia gravis.

a 15 unique families of children with monoADS reported 14 comorbidities among siblings of the participant; 13 reported one comorbidity; two families reported two comorbidities. Eight unique families of children with MS reported health conditions among siblings of the participant; seven reported one health condition and one reported two health conditions.

b 57 unique sets of parents of children with monoADS reported health conditions; 43 reported one health condition, eight reported two health conditions, two reported three health conditions and three reported four health conditions; one reported five health conditions. 33 unique sets of parents of children with MS reported health conditions; 23 reported one health condition, seven reported two health conditions, one reported three health conditions and two reported four health conditions.

c 14 unique participants with MS reported one or more comorbidities; 12 participants each reported a single comorbidity; two participants reported two comorbidities.

d 25 unique participants with monoADS reported one or more comorbidities; 18 reported one comorbidity; five participants reported two comorbidities; two reported three comorbidities.

### MS cohort characteristics

Participants with MS were more likely to be female, born outside of Canada, older at the time of neurological onset, older at the time of HRQoL assessment, have shorter lengths of stay in hospital at disease onset, and have shorter lengths of follow-up when compared to those with monoADS (Table 1). None of the participants received corticosteroids within 30 days of HRQoL assessment.

Multiple HRQoL assessments were available for 52 of the 65 (80%) MS participants over 1.73 (0.42–3.77) years. At initial HRQoL assessment, the median (IQR; min-max) number of relapses was 1 (0–3; 0–8) and 35 of 65 (54%) participants with MS were receiving DMTs. During the course of HRQoL assessments, 11 participants with MS experienced relapses (ranging from one to four relapses per person) and 49 of the 65 (75%) MS participants were exposed to DMTs.

### Univariate and bivariate analyses

On univariate analysis, children with MS reported lower HRQoL (*p* < 0.01) when compared to those with monoADS. Parents of participants with MS also reported lower HRQoL and lower family functioning when compared to parents of children with monoADS (Table 1). Although random effects models are capable of evaluating both within- and between-participant differences in HRQoL, we observed relatively little change (median (IQR) change of 6.9 (4.0–9.8)) in HRQoL among participants in our dataset. There was insufficient within-participant variation to evaluate within-participant changes over time.

Unadjusted analyses (Table 3) showed associations between lower HRQoL of the child ( $HRQoL_{it}^{child}$ ) and lower HRQoL of the parent ( $HRQoL_{it}^{parent}$ ), an MS diagnosis, lower family functioning ( $HRQoL_{it}^{family\leftarrow parent}$ ), lower BSMSS Occupation Scores of the parents, more morbidities of the child and parent, and functional neurological impairments of the child.

**Table 3.** Bivariate analyses of associations between the child’s HRQoL and covariates.

Predictor	Beta coefficient (95% CI)	Test statistic	*p* value
Key predictor			
Parent’s HRQoL	0.26 (0.20, 0.32)	8.57	**<0.001**
Instrumental Variable			
Family’s HRQoL	0.26 (0.20, 0.32)	8.90	**<0.001**
Time-invariant predictors			
MS diagnosis	–6.36 (–10.26, –2.46)	–3.19	**0.001**
Child’s sex	–2.58 (–6.28, 1.24)	–1.32	0.17
Child’s age at neurological onset	–0.25 (–0.65, 0.14)	–1.25	0.21
Length of stay for first attack	–0.003 (–0.20, 0.19)	–0.03	0.98
Child born in Canada	–3.16 (–8.77, 2.44)	–1.11	0.27
BSMSS Occupation Score	0.20 (0.04, 0.36)	2.51	**0.01**
Number of children in the family	0.14 (–1.85, 1.57)	–0.16	0.88
Child’s comorbidities	–8.90 (–12.08, –5.72)	–5.48	**<0.001**
Health conditions of parents	–2.16 (–4.14, –0.19)	–2.15	**0.03**
Health conditions of siblings	–1.42 (–6.34, 3.50)	–0.56	0.57
Time-variant predictors			
Child’s age at questionnaire	–0.11 (–0.40, 0.19)	–0.71	0.48
Functional neurological impairments	–4.29 (–8.19, –0.39)	–2.15	**0.03**

HRQoL: health-related quality of life; CI: confidence interval; MS: multiple sclerosis; BSMSS: Barratt Simplified Measure of Social Status. *P* values < 0.05 were considered statistically significant and presented in bold text.

### Multivariable analyses

When we estimated the child’s HRQoL adjusting for covariates, but not parental HRQoL, the diagnosis of MS was associated with lower HRQoL of the child (Table 4; Model 1**)**. Lower parental HRQoL was also associated with the diagnosis of MS (Table 4; Model 2). When we estimated the child’s HRQoL adjusting for covariates, but not the diagnosis of MS, lower HRQoL of the child was associated with lower HRQoL of the parents (Table 4; Model 3). Finally, when we estimated the child’s HRQoL adjusting for covariates, parental HRQoL, and the diagnosis of MS, the child’s HRQoL remained associated with the HRQoL of the parents, but the child’s HRQoL was no longer associated with the diagnosis of MS (Table 4; Model 4).

**Table 4.** Adjusted beta coefficients (95% confidence intervals) for the associations between the listed outcomes and predictors after adjusting for covariates^a^.

Models	Outcome	Predictor(s)	Beta coefficient (95% CI)	*p* value	*R*^b^ overall
Model 1	Child’s HRQoL	MS Diagnosis	–6.38 (–10.72, –2.04)	**0.004**	0.15
Model 2	Parent’s HRQoL	MS Diagnosis	–9.91 (–14.72, –5.11)	**<0.001**	0.16
Model 3	Child’s HRQoL	Parent’s HRQoL	0.33 (0.24, 0.43)	**<0.001**	0.26
Model 4	Child’s HRQoL	Parent’s HRQoL	0.32 (0.22, 0.41)	**<0.001**	0.27
		MS Diagnosis	–3.16 (–7.27, 0.95)	0.13	

CI: confidence interval; HRQoL: health-related quality of life; MS: multiple sclerosis. *P* values < 0.05 were considered statistically significant and presented in bold text.

a All four models were adjusted for sex, age at onset, length of stay in hospital for first attack, born in Canada, BSMSS Occupation Score, number of siblings, comorbidities of the participant, health conditions of the participant’s parents, and health conditions of the participant’s siblings, participant’s age at the time of HRQoL assessment, and presence of functional neurological impairments.

b Model 1 showed that the diagnosis of MS was associated with lower HRQoL of the child when we adjusted for covariates, but not parental HRQoL. The standardized beta coefficient for MS Diagnosis in Model 1 is the direct effect of the diagnosis on the child’s HRQoL (c’ in Figure 1). Model 2 showed an association between the diagnosis of MS and lower parental HRQoL (the mediator). The standardized beta coefficient for the MS Diagnosis in Model 2 is the partial indirect effect of the diagnosis on the child’s HRQoL (a in Figure 1). Model 3 showed that lower parental HRQoL (the mediator) was associated with lower HRQoL of the child. The standardized beta coefficient for the Parent’s HRQoL in Model 3 is the partial indirect effect of the diagnosis on the child’s HRQoL (b in Figure 1). Finally, we observed that the association shown in Model 1 between the diagnosis of MS and the child’s HRQoL diminished after adjusting for parental HRQoL (Model 4).

We did not observe an interaction between parental HRQoL and study visit number.

## Discussion

We found that parental HRQoL mediated the relationship between the diagnosis of MS and the HRQoL of affected children. The diagnosis of MS (versus monoADS) related to lower HRQoL among parents, which in turn related to lower HRQoL among affected children. Notably, the HRQoL scores among the comparator group (children with monoADS) were similar to those of published normative cohorts; our findings suggest that children with MS might report HRQoL scores similar to those of children with monoADS or even healthy children if their parents are able to attain sufficiently high HRQoL.^28^ These findings demonstrate the importance of accounting for parental HRQoL when evaluating the HRQoL of children with MS. Our findings are consistent with prior reports of mediation or associations between parental depression, parental distress, and parental perceptions of social support and the HRQoL of children with IBD or fecal incontinence.^8,29,30^

Our cohort of children with MS who experienced few relapses and minimal persistent neurological impairments allowed us to inform on the broader effects of an MS diagnosis on wellbeing independent of these factors. Understanding of the psychological burden of an MS diagnosis is increasingly relevant as children and adults with MS experience improved disease control in the context of improved DMTs. We might have observed a stronger association between MS diagnosis and HRQoL in pediatric-onset MS cohorts with greater relapse frequency and neurological impairments. Our observation of negligible within-participant change in HRQoL over time is consistent with longitudinal HRQoL observations among adults with MS; however, longer periods of observation might identify factors that influence HRQoL changes within children over time.^31^

Future studies are needed to identify factors that influence the HRQoL of parents of children with MS. We did not observe a difference in the number of family health conditions between MS and monoADS participants; however, we previously observed a higher prevalence of physical conditions and of any mood or anxiety disorder among mothers of children with MS when compared to mothers of children without MS.^32^ Qualitative interviews with parents whose children live with MS in the United Kingdom revealed that parents’ experiences with MS were dominated by feelings of uncertainty, including daily uncertainty, interaction uncertainty (related to uncertainty about how to respond to skepticism about the child’s MS diagnosis), and future uncertainty.^5^ Evaluation of factors that associate with the HRQoL of parents should therefore consider parental health conditions, perceived parental uncertainty, and parental worry.

Our study has several strengths. We used the PedsQL^TM^, which has been widely used to evaluate HRQoL in children with chronic health conditions. We accounted for multiple factors that can influence HRQoL in children and youth, including sociodemographic factors and comorbidities. Our study design benefited from repeated measures using random-effects specifications, which allowed for improved estimation of variance compared to cross-sectional methodologies.

Study limitations should be considered. We did not capture which parent responded to the Family Impact Module at each visit, which prohibited us from informing on between-parent differences. We did not, however, observe an association between parental HRQoL and study visit number when estimating the child’s HRQoL, suggesting that either the parent respondent did not change across observations or both parents had similar responses. Our adjustment for the number of parental health conditions (both parents combined) when estimating the child’s HRQoL reflects prior literature that suggests that parental illness associates with the child’s HRQoL.^20^ However, combining the comorbidities of both parents may not have optimally addressed confounding of the child-parent HRQoL relationship for the reporting parent. We found an association between the overall HRQoL of parents and children. Future studies of associations between the subscale or dimensional scores of children with MS and their parents are needed to better understand this relationship and evaluate how parental characteristics influence this relationship to tailor targeted interventions for parents. The study inclusion criteria changed over time, reflecting changes in study objectives due to three independent grant funding cycles during participant enrollment. We do not expect that this protocol change affected the study findings because we adjusted for age at disease onset.

Our findings implicate parental HRQoL as a target to improve the HRQoL of parents and children with MS. Studies of families of pediatric cancer survivors report that transmission of parental psychological distress to children may be modified by psychosocial family risk, whereby interventions focused on positive beliefs about the future, a positive family environment, high levels of social support, and low levels of family problems buffer the association between parental psychosocial distress and child HRQoL.^33,34^ Identifying factors that affect the association between parent psychological distress and child HRQoL may provide insight into developing targets for psychosocial interventions.

## Supplemental Material

sj-docx-1-msj-10.1177_13524585211061521 – Supplemental material for The health-related quality of life of children with multiple sclerosis is mediated by the health-related quality of life of their parents Click here for additional data file. Supplemental material, sj-docx-1-msj-10.1177_13524585211061521 for The health-related quality of life of children with multiple sclerosis is mediated by the health-related quality of life of their parents by Julia O’Mahony, Brenda Banwell, Audrey Laporte, Adalsteinn Brown, Lady Bolongaita, Amit Bar-Or, E Ann Yeh and Ruth Ann Marrie in Multiple Sclerosis Journal

sj-docx-2-msj-10.1177_13524585211061521 – Supplemental material for The health-related quality of life of children with multiple sclerosis is mediated by the health-related quality of life of their parents Click here for additional data file. Supplemental material, sj-docx-2-msj-10.1177_13524585211061521 for The health-related quality of life of children with multiple sclerosis is mediated by the health-related quality of life of their parents by Julia O’Mahony, Brenda Banwell, Audrey Laporte, Adalsteinn Brown, Lady Bolongaita, Amit Bar-Or, E Ann Yeh and Ruth Ann Marrie in Multiple Sclerosis Journal
