# Supplementary material for: The health-related quality of life of children with multiple sclerosis is mediated by the health-related quality of life of their parents
Source: Mult Scler. 2022 Feb 7;28(8):1299–310. doi: 10.1177/13524585211061521 (PMC9189724; doi:10.1177/13524585211061521)
Supplement: sj-docx-1-msj-10.1177_13524585211061521 – Supplemental material for The health-related quality of life of children with multiple sclerosis is mediated by the health-related quality of life of their parents [file sj-docx-1-msj-10.1177_13524585211061521.docx]

| **First names (will be abbreviated on Pubmed)** | **Surnames (not abbreviated)** | **Institution** | **Highest Degree** |
| --- | --- | --- | --- |
| Mark | Awuku | University of Windsor | MD |
| J Burke | Baird | McMaster University | MD |
| Virender | Bhan | Dalhousie University | MD |
| David | Buckley | Janeway Children's Health and Rehabilitation Centre | MD |
| David | Callen | Hamilton Health Sciences Center | MD |
| Mary B | Connolly | Children’s Hospital of British Columbia | MBBCh |
| Marie-Emmanuelle | Dilenge | Montreal Children’s Hospital | MD |
| Asif | Doja | Children’s Hospital of Eastern Ontario | MD |
| Simon | Levin | University Hospital  London | MD |
| Anne | Lortie | L’Hopital St. Justine | MD |
| E Athen | MacDonald | Hotel-Dieu | MD |
| Jean K | Mah | Alberta Children’s Hospital | MD |
| Brandon | Meaney | Hamilton Health Sciences Center | MD |
| David | Meek | Saint John Regional Hospital Facility | MD |
| Daniela | Pohl | Children’s Hospital of Eastern Ontario | MD |
| Giullaume | Sebire | Montreal Children’s Hospital | MD |
| Sunita | Venkateswaran | Children’s Hospital of Eastern Ontario | MD |
| Amy | Waldman | Children’s Hospital of Philadelphia | MD |
| Katherine | Wambera | Victoria General Hospital | MD |
| Ellen | Wood | Dalhousie University | MD |
| Jerome | Yager | Children’s Stollery Hospital | MD |

Appendix 1: Network List
