# Supplementary material for: The health-related quality of life of children with multiple sclerosis is mediated by the health-related quality of life of their parents
Source: Mult Scler. 2022 Feb 7;28(8):1299–310. doi: 10.1177/13524585211061521 (PMC9189724; doi:10.1177/13524585211061521)
Supplement: sj-docx-2-msj-10.1177_13524585211061521 – Supplemental material for The health-related quality of life of children with multiple sclerosis is mediated by the health-related quality of life of their parents [file sj-docx-2-msj-10.1177_13524585211061521.docx]

Appendix 2: Supplementary Methods

### PedsQL^TM^

Participants and their parents were mailed the paper versions of the PedsQL^TM^ questionnaires two weeks before their in-person visits and were instructed to complete the questionnaires at home when alone. Children received the version of the PedsQL^TM^ Inventory (Child Report) that corresponded to their age at the time of reporting. Parent proxy reports were not required because all participants were >5 years at the time of HRQoL assessment. Participants were instructed to return the completed questionnaires during in-person visits in sealed envelopes provided to them. Participants who did not complete questionnaires before clinic visits were asked to complete them in clinic in a private area.

### Barratt Simplified Measure of Social Status (BSMSS) Occupation Score

Parental occupations were queried in one of two ways. Between August 2015 and January 2018, parents selected their occupations from the BSMSS list of occupations. For participants who were lost to follow-up prior to August 2015, a single scorer (JO’M) ascribed an occupation from the BSMSS list of occupations to each parent using the self-reported parental occupations captured during structured interviews with a trained genetic counsellor that were performed during the second study visit (3 months post-onset).

Parental occupations for the current analysis were ascertained using the BSMSS occupations list for 92 (44%) families and the genetic interview for 115 (56%) families. Reliability between these two methods was assessed by analyzing the subset of 56 participants who reported their occupation via the BSMSS and genetic interview using intraclass correlation coefficients (ICCs) and their 95% confidence intervals (CI) based on average-measures, absolute agreement, two-way mixed effects model. Among the sub-set of parents who reported their occupations via the genetic-interview and BSMSS list of occupations, IRR was good [ICC (95% CI) was 0.68 (0.45-0.81)].

### Comorbidities and Health Conditions

Comorbidities of the participant and familial health conditions were captured in two ways. At enrollment, parents and participants (when age appropriate) were asked to report all familial diagnoses; responses were recorded on CRFs and changes were noted at follow-up study visits. Additionally, parents of participants enrolled between September 2004 and May 2013 were offered a structured interview with a trained genetic counselor at their second study visit (3 months post-neurological onset), at which time familial health conditions were recorded.

Reported comorbidities and health conditions were reviewed by two scorers (JO’M and BB) and included in this analysis if they are listed on the comorbidity questionnaire, which is validated for use in the adult MS population and modeled after the self-report comorbidity questionnaire by Sangha et al. A comorbidity count was utilized because severity data were unavailable. Parental comorbidities were combined for the purposes of the current analyses because the identities of the parents who completed the HRQoL questionnaires were unknown.

After reviewing all reported diagnoses, JO’M and BB noted ten reported diagnoses (Table 2) that were not captured by the comorbidity questionnaire, but were considered by JO’M and BB to warrant consideration for the current analyses. Expanded comorbidity and health condition scores were therefore assigned to each participant, their siblings, and parents that included these ten additional diagnoses. One of the ten diagnoses, MS, has been shown to impact HRQoL among affected adults, but was not included in the comorbidity questionnaire because the questionnaire was designed specifically for people diagnosed with MS. Autism spectrum disorder (ASD) was not included in the comorbidity scale optimized for adults with MS, but we include it in our comorbidity list as we feel that it has an impact on family functioning. Eight other diseases [tics, Guillain-Barré Syndrome (GBS), endocrine disorder, Parkinson’s disease, central nervous system (CNS) aneurysm, myasthenia gravis (MG), sarcoidosis, and Usher’s syndrome] were also included. These ten additional diagnoses are herein referred to as ‘expanded conditions’.

Among the 207 participants, 172 (83%) completed the genetic interview. To address potential biases in data collection (genetic interview versus CRF) and the expanded comorbidities and health conditions scores, two sub-analyses were performed. First, only diagnoses captured via CRF (and not the genetic interview) were modeled because all participants had the opportunity to complete the CRF while only a sub-sample completed both the CRF and the genetic interview. Second, only diagnoses captured by the comorbidity scale (and not the expanded conditions) were modeled.

Two sub-analyses were performed to evaluate potential biases due to the expanded comorbidities score and the methods of capturing comorbidities (genetic interview versus CRF). The independent variables found to be statistically significant in the ${HRQoL}_{it}^{child}$model (${HRQoL}_{it}^{parent}$ and comorbidities of the affected child) remained consistent when the comorbidities score was limited to only the health conditions listed on the comorbidities scale and when the comorbidities were limited to those captured by the CRF. When the ${HRQoL}_{it}^{parent}$was modeled with the health conditions limited to the comorbidity scale (excluding the expanded conditions), the child’s comorbidities were no longer statistically significant (p=0.05). When the ${HRQoL}_{it}^{parent}$was modeled with the comorbidities limited to those captured by the CRF, the results remained consistent with the original model.

The original comorbidity scale captured 184 of the 206 (89%) reported health conditions, excluding the 22 expanded conditions. Notably, the genetic interview captured 78 (38%) diagnoses that were not reported via the CRF. The 35 families who did not complete the genetic interview reported a total of 28 health conditions, which may be an underestimate of their burden of disease.
